# Fabrication of Sub-Micron Polymer Waveguides through Two-Photon Polymerization in Polydimethylsiloxane

**DOI:** 10.3390/polym12112485

**Published:** 2020-10-26

**Authors:** Giulia Panusa, Ye Pu, Jieping Wang, Christophe Moser, Demetri Psaltis

**Affiliations:** 1 Optics Laboratory (LO), École Polytechnique Fédérale de Lausanne (EPFL), 1015 Lausanne, Switzerland; giulia.panusa@epfl.ch (G.P.); jieping.eth@outlook.com (J.W.); demetri.psaltis@epfl.ch (D.P.); 2 Laboratory of Applied Photonics Devices (LAPD), École Polytechnique Fédérale de Lausanne (EPFL), 1015 Lausanne, Switzerland; christophe.moser@epfl.ch

**Keywords:** two-photon polymerization (2PP), femtosecond, direct laser writing (DLW), photoinitiator (PI), divinylbenzene (DVB), polydimethylsiloxane (PDMS), polymer optical waveguides, digital holographic interferometry, self-focusing

## Abstract

Flexible ultra-compact low-loss optical waveguides play a vital role in the development of soft photonics. The search for suitable materials and innovative fabrication techniques to achieve low loss long polymer optical waveguides and interconnects has proven to be challenging. In this paper, we demonstrate the fabrication of submicron optical waveguides in polydimethylsiloxane (PDMS) using divinylbenzene (DVB) as the photopolymerizable monomer through two-photon polymerization (2PP). We show that the commercial oxime ester photoinitiator Irgacure OXE02 is suitable for triggering the DVB photopolymerization, resulting in a stable and controllable fabrication process for the fabrication of defect-free, 5-cm long waveguides. We further explore a multi-track fabrication strategy to enlarge the waveguide core size up to ~3 μm for better light confinement and reduced cross-talk. In these waveguides, we measured a refractive index contrast on the order of 0.005 and a transmission loss of 0.1 dB/cm at 710 nm wavelength.

## 1. Introduction

Optical waveguides are one of the most important photonic components indispensable in many of today’s technologies [1,2]. The development of new materials and novel fabrication technologies for the realization of optical waveguides have attracted great attention. In particular, polymer-based waveguides are crucial soft photonic building blocks [3,4,5] for the development of complex multifunctional platforms, such as chip-to-chip interconnects in electronic systems [6,7], lab-on-chips [8], optofluidic platforms [9], biomedical sensing [10,11], wearable physiological monitoring [12], and optogenetics [13,14,15]. Single-mode optical waveguides have been fabricated through direct laser writing (DLW), soft lithography, and thermal curing methods using poly-siloxane and other commercially available polymer materials [16,17,18,19]. Polydimethylsiloxane (PDMS) is an elastomer of fundamental technological importance; because of its physical and chemical properties, and because of its easy handling. It is widely used for the realization of optical waveguides by combining hot embossing and standard soft lithography processes [20,21,22]. These methods are generally constrained to the fabrication of two-dimensional structures. However, the implementation of miniaturized photonic components with increased integration density calls for new, versatile fabrication technologies, which enable the formation of arbitrary submicron 3D shapes within a bulk material, such as two-photon DLW.

Multi-photon polymerization (MPP) is one of the most important technological achievements for the microfabrication of fine structures in polymeric materials, because of its nonlinear feature resulting in a precisely localized and highly confined material modification [23]. Typically, polymer formulations for photopolymerization applications comprise a polymerizable component (the monomer) and a photosensitive component (the photoinitiator). Developments in these two-component materials are central to the field of MPP.

We have recently shown the fabrication of sub-2 µm waveguides in polydimethylsiloxane (PDMS) through multi-photon DLW without a photoinitiator (PI) [24]. The PI-free fabrication process using phenylacetylene as the monomer achieved an excellent refractive index contrast of 0.06 and a very low optical loss of 0.03 dB/cm in the 650–700 nm band. While the high refractive index contrast is clearly a result of a high degree of polymerization, the existence of long conjugated π-bonds in the polymer result in very poor transmission in wavelengths shorter than 600 nm. Furthermore, this process was subject to defects in the fabrication of waveguides longer than one cm due to material damage, which is likely a result of the uncontrollable beam collapse caused by the self-focusing of light upon photopolymerization [25].

In a photoinduced polymerization process, the reaction probability of polymerization is proportional to a power function of the laser intensity depending on the order of the nonlinearity involved. This creates an intensity-dependent refractive index profile during the laser irradiation, which causes self-focusing and beam self-trapping. Different from nonlinear refractive indices of electronic and molecular origin, this chemically originated nonlinear refractive index reacts rather slow, possibly at the millisecond time scale as limited by the monomer diffusion time and is rather large in magnitude. This self-focusing process is a direct function of the refractive index change between the pre- and post-polymerization state, which is dependent on the degree of polymerization and the monomer concentration. The self-focusing that leads to beam collapse and material damage is an intrinsic effect that needs to be taken into consideration in the waveguide writing process. Motivated by the need to minimize self-focusing, we explored other monomer/PI combinations that can lead to different characteristics in PDMS. In particular, styrene and its derivatives are intriguing as a monomer because of the lack of long-chain conjugated π-bonds in the resulting polymer, potentially resulting in a broader transmission band. Furthermore, owing to the continuing efforts in the development of functional PIs [26,27,28], the fabrication could benefit from new PIs specifically optimized for two-photon absorption [27].

In this work, we demonstrate the fabrication of long, submicron-sized, largely defect-free optical channel waveguides in PDMS using divinylbenzene (DVB) monomer and a commercial PI, Irgacure OXE02. In addition to the multi-cm waveguide length without defects, the transmission is broadband, limited only by scattering. The trade-off in achieving this, is a lower refractive index contrast in the waveguide fabricated, which results in higher transmission loss and less confined light compared with the PI-free process. Through this approach, we were able to fabricate single-track (simple) waveguides of ~0.5 µm width, which has a nearly flat loss of 13 dB/cm over the spectral range of 535–679 nm and is subject to crosstalk when placed close to one another in parallel. To improve the light confinement, we also used a multi-track (compound) waveguide strategy to increase the waveguide width. Characterizations in the compound waveguides show an optical loss of 0.1 dB/cm in the 710/10 nm spectral band, where we follow the convention of central wavelength/bandwidth, and a refractive index contrast of ~0.005.

## 2. Materials and Methods

### 2.1. Chemical Scheme

We tested a wide range of PIs and identified the commercial Irgacure OXE02 (BASF, Münster, Germany) [27] to be suitable for initiating the photopolymerization of DVB through two-photon absorption in our experiments. Furthermore, waveguides fabricated based on styrene were frail and did not sustain the solvent process to remove unreacted monomer and PI, possibly due to a small molecular weight in the polymer product and the lack of crosslinking. Therefore, we chose DVB as the monomer to promote crosslinking and address the stability issue.

**Figure 1:**
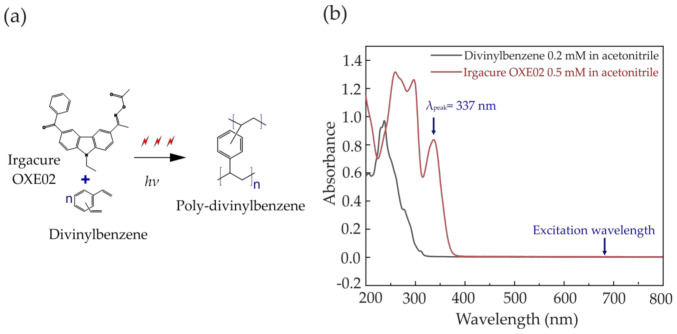
(**a**) Molecular structure of the divinylbenzene (DVB) monomer and Irgacure OXE02 and the polymerization of the monomer upon laser irradiation; (**b**) Absorption spectrum of 0.2 mM DVB and 0.5 mM Irgacure OXE02 in acetonitrile. Blue arrows indicate the absorption wavelength peak of the photoinitiator (PI) (337 nm) and the excitation wavelength of our system tuned to 680 nm.

The chemical scheme of the multiphoton polymerization is illustrated in Figure 1a. The UV-visible absorption spectra of DVB and OXE02 was measured using a UV-visible spectrometer (Cary-100, Agilent Technologies, Santa Clara, CA, USA) as shown in Figure 1b. The absorption bands of both reactants fall in the 200–400 nm spectral range, which fall in the spectral transparency band of the host PDMS [29]. Upon multiphoton absorption, Irgacure OXE02 initiates the polymerization of DVB, forming a cross-linked polymer. Compared with self-initiated photopolymerization in phenylacetylene [24], the presence of the PI in this reaction likely results in a lower polymer molecular weight due to frequent termination by combination [30].

### 2.2. Materials

All chemicals were used as purchased without further purification. DVB (80% purity, CAS Number 1321-74-0) was purchased from Sigma Aldrich (Buchs, Switzerland). Irgacure OXE02 (CAS Number 455-590-6) was requisitioned from BASF. Optically transparent biomedical-grade PDMS (NuSil MED6215, *n* = 1.406) was purchased from NuSil Technology (Carpinteria, CA, USA).

### 2.3. Sample Preparation

We used an optically clear, medically approved silicone elastomer, NuSil MED6215, for all our samples, which is widely used for human implantation devices [31] and thus suits our development goal well. It consists of a Part A (refractive index *n* = 1.409, viscosity *η* = 5.600 cP) and a Part B to be combined in a ratio of 10:1 followed by a mixing and defoaming process. To ensure the homogeneity of the PDMS substrate, which is the key to high-quality waveguides, the mixing and defoaming were performed three consecutive times in a Thinky automatic mixer for about 3 min each time, followed by a final deaeration procedure in a vacuum desiccator. We cured the PDMS samples at room temperature over 48 h to ensure surface flatness and parallelism with the working plane. For the optical clarity of the PDMS substrate, the above preparation was performed in an ISO7 cleanroom.

**Figure 2:**
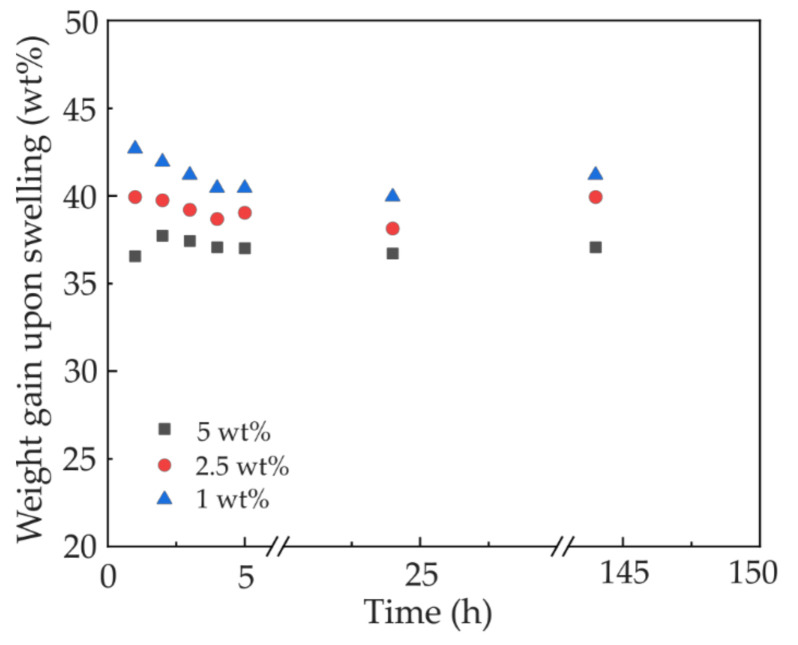
Weight gain upon swelling of pristine cured polydimethylsiloxane (PDMS) slabs, for 1, 2.5, and 5 wt% of Irgacure OXE02 in the DVB monomer.

We prepared 1-mm thick pristine PDMS slabs of 1 × 5 cm^2^ surface area and immersed them in the monomer-PI solution for 144 h to load the monomer and PI molecules into the PDMS intermolecular space. This step was significantly longer than our previous work [24], because the PI molecule is much larger than the monomer and takes a much longer time to permeate the PDMS slab and reach the saturation level. Over the course of this infiltration, we measured the evolution of the weight gain of the PDMS slabs for different concentrations of the PI in the DVB monomer, which is shown in Figure 2. Although the weight gain does not change much after the first two hours, quality waveguides could only be fabricated after 144 h of immersion, which clearly reveals the effect of the larger PI molecule. In all three PI concentrations, the weight gain reaches its peak in the first one or two hours, which is an indication of the fast mobility of the small monomer DVB molecules. The weight gain slightly drops in the subsequent few hours possibly due to the leaching of certain small molecules originally presented in the PDMS since DVB is a good solvent. In the succeeding hours, the weight gain slowly increases again, suggesting the slow infiltration of the much larger PI molecules into the PDMS. During the infiltration, a certain number of the PI molecules may be blocked on the surface of the PDMS slab, causing a hindrance for further material infiltration and resulting in lower weight gain in the sample with higher PI concentration.

### 2.4. Waveguide Fabrication

The general procedure of the waveguide fabrication follows that described in [24]. Based on a fabrication platform for DLW [24], which contains a mechanical motion stage with 5 cm travel range, we further integrated two high-precision piezo stages (Newport Spectra Physics GmbH NPX400SG-D and NPO250SG-D, Darmstadt, Germany) for accurate positioning and a tip-tilt-rotation stage to ensure the track parallelism with the slab surface over a distance of 5 cm.

Before the commencement of the writing process, we assembled the PDMS slab between a microscope slide and a glass coverslip, which was mounted on the tip-tilt-rotation stage. We performed careful alignment with the tip-tilt-rotation stage by focusing the laser beam on the water–glass interface and monitored the focus status in the reflection with a camera while moving one end to the other end of the tracks. This focus also serves as a location reference for positioning the laser focus inside the PDMS slab using the piezoelectric stage.

We focused a laser beam from a femtosecond Ti:Sapphire laser (Coherent Chameleon Ultra II, Santa Clara, CA, USA) at 80 MHz repetition rate and 140 fs pulse width into the PDMS slab sample with a high NA (0.7), long working distance, and water-immersion objective. In order to trigger the two-photon polymerization reaction, the laser was tuned to 680 nm to reach the absorption band of Irgacure OXE02 via two-photon absorption. The average beam power used in the fabrication ranges between 60 and 85 mW, and thus the peak intensity can be adjusted between ~1.93 and ~2.74 × 10^12^ W/cm^2^ based on an aberration-free, self-focusing-free focal spot. We refer to this intensity as the nominal writing intensity in later sections. During the laser writing, the PDMS slab proceeded at a predetermined constant velocity, while the lateral position of the focus was controlled by the piezoelectric stages. Upon the completion of the waveguide writing, we finished the sample with a 24-h ethanol bath to remove unreacted monomer and the PI.

### 2.5. Waveguide Characterization

We characterized the refractive index contrast Δ*n* of the poly-DVB waveguides using an interferometric imaging system [24]. Briefly, we illuminated the waveguides perpendicularly with a collimated beam from an He-Ne laser (λ = 632.8 nm) and imaged the waveguides on a complementary metal–oxide–semiconductor (CMOS) digital camera in a 4f imaging system incorporating a 20×, 0.42 NA objective. A plane wave reference beam was introduced and interfered with the imaging wave front at a predetermined angle to create an off-axis hologram. The phase change that the illumination laser beam undergoes passing the waveguide region reveals the refractive index contrast given the known waveguide dimensions, which was extracted from the numerically reconstructed object wave front.

The transmission loss (TL) measurements were performed by coupling broadband light from a thermal light source into the waveguides [24]. We used a 50×, 0.8 NA objective for focusing the light at the input end of the waveguide being tested, and a 20×, 0.42 NA objective for imaging the waveguide output on a CMOS camera by means of a 4f imaging system. The TL measurements were carried out through the cut-back method, where we measured the ratio of the output power from the same waveguides cut into two different lengths, *L*_1_ and *L*_2_.This ratio indicates the optical loss due to material absorption and light scattering caused by inhomogeneous waveguide walls and polymer density fluctuation. To obtain better statistics, we measured three different isolated waveguides fabricated under identical parameters in each type of both simple and compound waveguides. The transmission loss was measured at several spectral points using a number of color filters right after the light is coupled into the waveguides. We calculated the transmission loss in dB/cm for different wavelength regions following the formula:

(1) $$TL=10{log}_{10}(P_{ratio})\Delta L$$

where $P_{ratio}=\frac{P_{1}}{P_{2}}$, Δ*L = L*_1_ − *L*_2_, and *P*_1_ and *P*_2_ are the measured waveguide output power at lengths *L*_1_ and *L*_2_, respectively.

## 3. Results

### 3.1. Simple Waveguides

**Figure 3:**
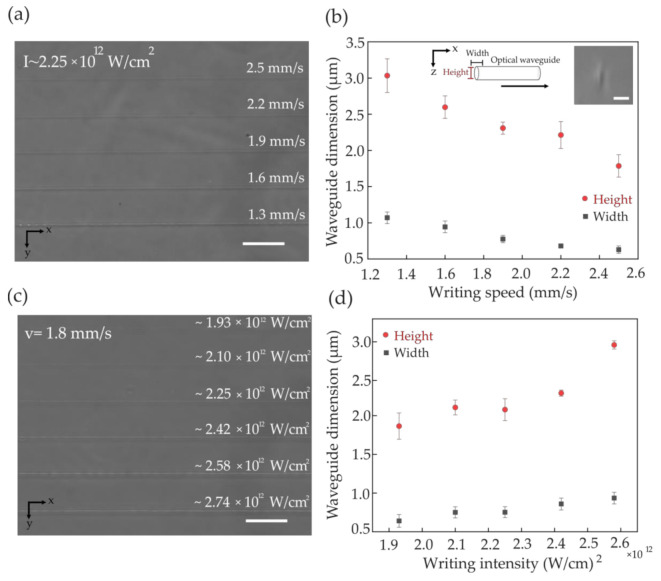
(**a**) Phase-contrast microscopy top view image of the recorded poly-DVB optical waveguides written at constant peak intensity I ~ 2.25 × 10^12^ W/cm^2^ and varying fabrication speed, ranging from 1.3 to 2.5 mm/s; (**b**) Waveguide width and height evolution as a function of increasing writing speed where the error bar derives from the measurement of three waveguides; the inset shows the cross-sectional view of a single-track waveguide where the scale bar measures 5 μm; (**c**) and (**b**) display the same as above as a function of increasing fabrication peak intensity from 1.93 to 2.74 × 10^12^ W/cm^2^ and constant speed v = 1.8 mm/s. Error bars indicate the standard deviation derived from the measurement of five different waveguides geometry. Scale bars dimension in (**a**) and (**c**) is 50 μm.

Finding the right combination of the various fabrication parameters is a crucial challenge in DLW photopolymerization, requiring a compromise between the irradiating laser intensity and writing speed in order to avoid material damage. We wrote waveguides over 5 cm long samples, isolated from each other by an inter-waveguide distance of about 500 μm to eliminate any crosstalk. For each set of parameters, we fabricated five waveguides, from which three were interrogated for statistics. We investigated the finished samples of isolated single waveguides (simple waveguides) using a phase-contrast microscope (Olympus IX-71, Hamburg, Germany) to verify the formation of the poly-DVB waveguides embedded in the PDMS framework (Figure 3a,c). The cross-section of single-pass laser tracks (see the inset in Figure 3b for a typical example) was measured to be ~0.6–1 μm wide and ~1.5–3 μm high depending on the writing parameters, as shown in Figure 3b,d. The dimensions (the width and the height) of the focal volume during the fabrication procedure depend not only on the laser pulse energy and the numerical aperture of the writing objective but also on the writing speed. Figure 3a,b reveals the evolution of the waveguide geometry (width and height) as a function of increasing writing speed at a constant writing laser intensity (I ~ 2.25 × 10^12^ W/cm^2^). Figure 3c,d shows the waveguide geometry as a function of increasing laser intensity at a constant writing speed v = 1.8 mm/s. Overall, both the axial and lateral dimensions of the waveguide structure depend linearly on laser peak intensity and writing speed, showing an aspect ratio of about 1:3; nevertheless, experimental sample variations such as monomer/PI solution infiltration efficiency, and optical settings during the writing, strongly affect the waveguide fabrication where experimental conditions remain unchanged. This reflects in small geometrical fluctuations in the waveguide size throughout different samples fabricated with the same experimental parameters such as laser focal intensity and writing speed. This can be observed in the plot in Figure 3d.

**Figure 4:**
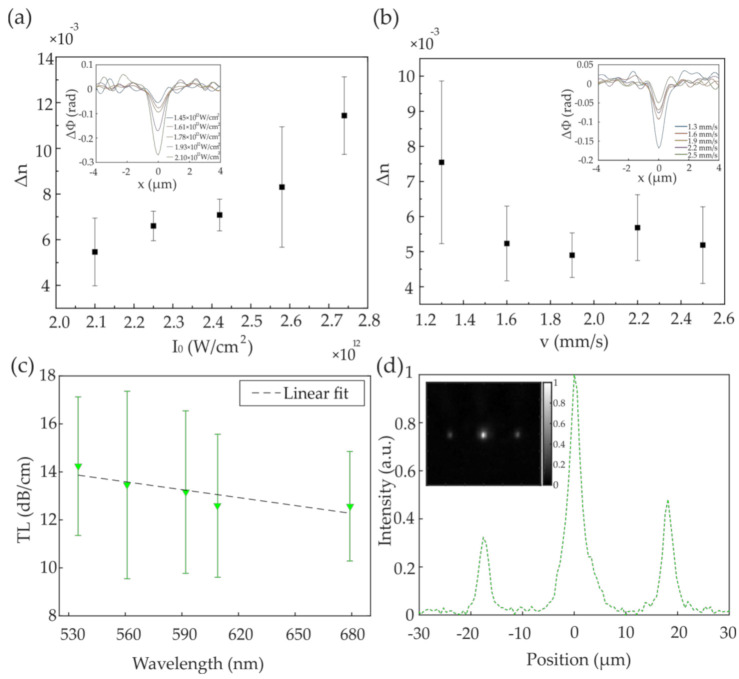
(**a**) Evolution of the refractive index as a function of increasing peak intensity (and constant writing speed v = 1.8 mm/s) is displayed. The laser peak intensity ranged between ~2.10 × 10^12^ W/cm^2^ and ~2.74 × 10^12^ W/cm^2^; (**b**) Refractive index contrast for waveguides recorded at ~2.25 × 10^12^ W/cm^2^ constant laser intensity and different writing speed, increasing from 1.3 to 2.5 mm/s. The insets display the measurement of the phase profile for different laser peak intensities and velocities, where each phase profile is the mean profile measured from three waveguides written with the same fabrication parameters; (**c**) Transmission loss in dB/cm as a function of wavelength, where colored filters have been inserted in the optical path to measure the output from three different simple waveguides written 500 μm apart from each other; (**d**) Output intensity profile of three simple waveguides at an inter-waveguide distance of ~18 μm; light in the 535/43 nm spectral band has been coupled in the central waveguide. Waveguide outputs are displayed in the image inset. Error bars indicate the standard deviation from three waveguides fabricated with the same experimental parameters.

We measured the refractive index contrast as a function of focal intensity at a constant writing speed and as a function of writing speed at a constant focal intensity, as shown in Figure 4a,b, respectively. Our experiments suggest that the polymerization threshold for DVB is about ~1.93 × 10^12^ W/cm^2^ for simple waveguides based on visual determination with phase-contrast microscopy. As we show in Section 3.2, this is higher than the actual polymerization threshold, and it is possible to write waveguides at a slightly lower intensity, since phase-contrast microscopy cannot detect the presence of low-concentration polymer in such a small volume with sufficient sensitivity. The refractive index change induced in the polymer material increases linearly from 0.005 to 0.012 with laser intensity in the 2.10–2.8 × 10^12^ W/cm^2^ range (Figure 4a). We observed a weaker effect of the writing velocity on the measured refractive index change between the polymer waveguides and the PDMS framework, as shown in Figure 4b, which is in good accordance with other works on photopolymerization [32]. At a constant laser intensity of ~2.25 × 10^12^ W/cm^2^ and 1.3 mm/s writing speed, we measured a refractive index change of ~0.0075, while at higher speeds the refractive index contrast stabilizes around 0.005. The insets in both Figure 4a,b show the mean phase profiles recorded along the waveguides in our interferometric system.

Due to the elliptic cross-section, the V-number and thus the modal properties of the simple waveguides are birefringent. Based on a Δ*n* of 0.005, the semi-major axis length of the elliptical core of about 1.5 µm and the manufacturer-specified PDMS cladding refractive index of 1.406, we thus estimated that the upper limit of the V-number for the simple waveguides is 2.1, at the shorter end of the tested spectral band (535 nm), based on the V-number definition for elliptical waveguides reported in [33]. The V-number may be slightly higher (<0.5% as estimated from Sylgard-184 data) when material dispersion is considered. At the longer end (679 nm), the V-number is 1.6; therefore, the simple waveguides are single-mode.

To show the crosstalk, we wrote simple waveguides with the same fabrication parameters (2.25 × 10^12^ W/cm^2^ and 2 mm/s), and tested them at an inter-waveguide spacing of 20 μm, which reduced to ~18 μm (inset in Figure 4d) after the solvent process to remove the unreacted monomer and PI. At this inter-waveguide spacing and 1 cm sample length, the simple waveguides present crosstalk, as shown in Figure 4d where the intensity profile along the waveguides is shown. The image inset shows the simple waveguides output in the 535/43 nm spectral band; at ~18 μm inter-waveguide spacing, the output light is clearly visible also from the two adjacent cores whereas only the central waveguide has been coupled. 

### 3.2. Compound Waveguides

In order to achieve long, defect-free waveguides we optimized the fabrication parameters for simple waveguides to be 1.78 × 10^12^ W/cm^2^ nominal intensity and 3.2 mm/s writing velocity. This intensity is lower than the visually-determined threshold of 1.93 × 10^12^ W/cm^2^ but significantly reduces the probability of defects. At this lower fabrication intensity, Δ*n* is further reduced and the crosstalk is more significant. Thus, the simple waveguides present a unique challenge of excessive crosstalk when being implemented in a high-density integration. Although increasing Δ*n* is the most effective way to minimize the crosstalk, it is constrained by the current photochemistry of choice, and more fundamentally, by the intrinsic self-focusing and beam collapse leading to material damage and fabrication defects.

**Figure 5:**
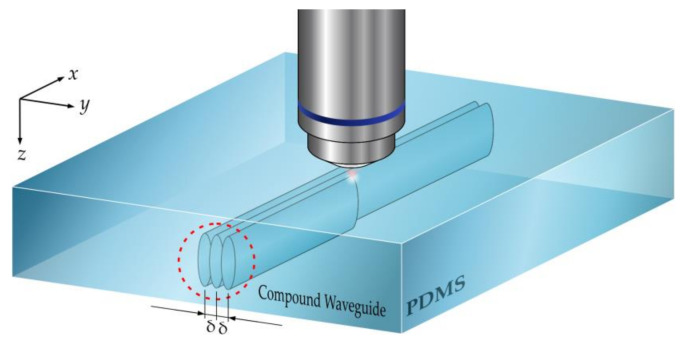
Schematic of the writing of compound waveguides. The PDMS sample is displaced by a small distance δ by means of a piezoelectric stage in the y direction. A long travel range mechanical stage moves the sample over centimeters in the x-direction.

Given the fundamental constraints and a goal of inter-waveguide spacing of 10 µm at the length of 5 cm, the only feasible approach is to increase the waveguide width for better mode confinement. Since this was difficult to achieve with larger focal spot size because of the required light intensity for multi-photon absorption, we instead fabricated compound waveguides where multiple tracks go side-by-side in parallel with a very small interval spacing δ (Figure 5). The spacing is so small that the potential barrier between the individual tracks is tunneled through easily and the multiple tracks effectively behave like a single, wider waveguide (a compound waveguide). This approach also calls for a lower fabrication intensity and a higher writing speed in order to avoid material damage caused by the refractive index modification carried by the adjacent core.

**Figure 6:**
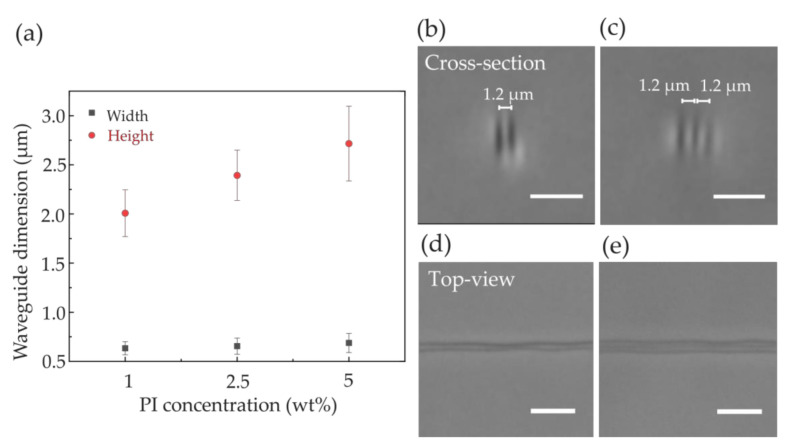
(**a**) Waveguide dimensions as a function of the Irgacure OXE02 concentration into the DVB monomer. The size of the waveguides increases with increasing PI concentration. The isolated, single waveguides were fabricated at an intensity of ~1.78 × 10^12^ W/cm^2^ and a velocity of 3.2 mm/s. Scale bars measure 5 μm; error bars indicate the standard deviation from five different waveguides; cross-sectional view taken in a phase contrast microscope (Nikon IX-71) of a double-cored (**b**) and a triple-cored (**c**) poly-DVB waveguides; top view image of double (**d**) and triple-track (**e**) poly-DVB waveguides.

The compound waveguides were fabricated with a nominal focal intensity of 1.78 × 10^12^ W/cm^2^ and a writing velocity of 3.2 mm/s, which were optimized from the fabrication of simple waveguides. We first fabricated short double- or triple-track compound waveguides at an intra-waveguide interval δ = 1.2 μm for a pilot study at three different PI concentrations and measured the dimensions of the individual constituent simple waveguide. At the same fabrication parameters, both waveguide width and height increase when a higher concentration of photoinitiator is used (Figure 6a). At 1 wt% PI concentration, the lateral and axial dimension of the waveguides were measured to be ~0.63 μm and ~2 μm on average, respectively, and reached ~0.69 μm and ~2.7 μm, respectively, at a PI concentration of 5 wt%. The cross-sectional and side view of the compound waveguides acquired from a phase contrast microscope is shown in Figure 6b–e.

**Figure 7:**
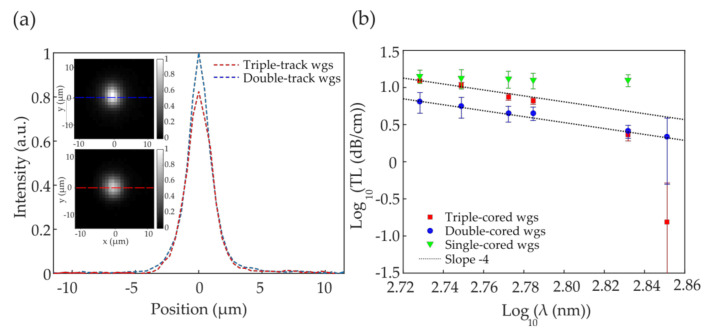
(**a**) Output intensity profile of a double-cored (blue) and triple-cored (red) waveguides (wgs), at 535/43 nm spectral band. Waveguide outputs are displayed in the image inset; (**b**) Transmission loss in dB/cm as a function of wavelength for simple (green), double- (blue) and triple- (red) track waveguides. Dot lines indicate the linear fit with −4.0 slope, for both double- and triple-track waveguides, typical of pure Rayleigh scattering.

We then fabricated 5-cm long, double- and triple-track compound waveguides at 5 wt% PI concentration and 1.2 μm intra-waveguide interval for more comprehensive characterization. The refractive index contrast Δ*n* of the compound waveguides was measured using the interferometric imaging system [24]. Both the double- and triple-track waveguides show a refractive index contrast on the order of Δ*n* = 0.005, calculated from the phase we extracted from the interferometric image of the waveguides. The V-number ranges from 2.22 down to 1.68 over the spectral band from 535 nm to 710 nm when we consider the waveguide cross-section as a square of side 2.7 µm. Figure 7a shows the waveguide output intensity profiles of a double-track (blue) and a triple-track (red) waveguide in the 535/43 nm spectral band, whose two-dimensional intensity distribution is shown in the inset. Figure 7b shows the transmission loss at the spectral points of measurement in a log-log fashion, where a straight line of a −4.0 slope would indicate a loss of pure Rayleigh scattering nature. In Figure 7b, the double-track and triple-track data fit well to lines of slope −3.8 and −7.1, respectively. Overall, in the double-track waveguides the TL drops from 6.51 dB/cm at 535/43 nm to 2.18 dB/cm at 710/10 nm, while in the triple-track waveguides it changes from 12.40 dB/cm to 0.15 dB/cm at the same two spectral points. The transmission loss for the different types of waveguides and in the different wavelength ranges is summarized in Table 1.

## 4. Discussion

To obtain further insight into the optical process in the photopolymerization in our experiments, we consider the polymerization kinetics [30], noting that the irradiation time is generally brief (on the order of sub-one ms) in our system and a steady state of the reaction is not reached. We also note from our measurements that the polymer concentration is low and highly localized in the writing region, implying that the monomer concentration remains nearly constant. The final concentration of the polymerized DVB, $c_{pDVB}\left(r\right)$ where r is the spatial coordinate, is directly proportional to the time-integration of the concentration of the PI radicals $c_{PI*}\left(r,t\right)$ during the laser irradiation, i.e., $c_{pDVB}\left(r\right)=\int \eta_{p}c_{PI*}\left(r,t\right)dt$, where $\eta_{p}$ is the polymerization coefficient depending on the initiation efficiency, propagation constant, termination constant, and monomer concentration of the specific PI-monomer combination.

The instantaneous concentration of the PI radicals generated through two-photon absorption during the laser irradiation is expressed as $\frac{\partial }{\partial t}c_{PI*}\left(r,t\right)=\sigma_{2}I^{2}\left(r-vt\right)c_{PI}\left(r,t\right)$; where $\sigma_{2}$ is the two-photon absorption cross-section of the photoinitiator molecule in Goeppert–Mayer units; $I\left(r\right)$ is the irradiation intensity expressed in terms of number of photons; v is the velocity of writing; and $c_{PI}\left(r,t\right)$ is the concentration of the remaining photoinitiator. Furthermore; due to the small region of writing, PI* is subject to a diffusion process as $\frac{\partial }{\partial t}n_{PI*}\left(r,t\right)=D_{PI*}\nabla^{2}n_{PI*}\left(r,t\right)$, where $D_{PI*}$ is the diffusion constant. The system is implicitly highly nonlinear, since the intensity $I\left(r-vt\right)$ at point r will be modulated by the refractive index profile $\Delta n_{pDVB}\left(r\right)$ it creates, which can be modeled with the unidirectional pulse propagation equation [34]

**Figure 8:**
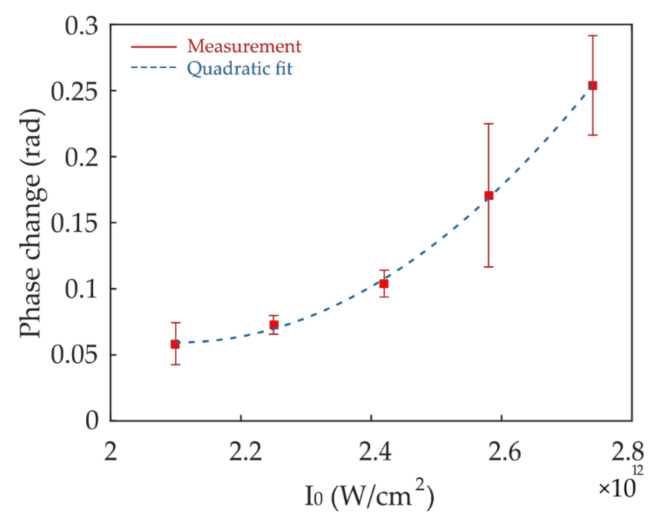
Plot of the measured phase change in the waveguides as a function of the writing intensity. The error bar indicates the standard deviation among three different waveguides. Dashed line shows a quadratic regression.

Heuristically, the diffusion equation in the coupled equation system causes c_PI*_ to expand spatially from the source term once the PI molecules are radicalized. In the one-dimensional writing of a waveguide, this expansion is two-dimensional in the height and width. Therefore, the refractive index profile of the fabricated waveguide is jointly determined by σ_2_ and D_PI*_. Regardless of the exact distribution of Δ*n_pDVB_* and the extent of the polymerization region, the total phase change as measured in the interferometric imaging system, where the width of the polymerized structure is below the diffraction limit, is $\Delta \Phi =k\Delta n_{pDVB}h\propto I_{0}^{2}$, where $k=\frac{2\pi }{\lambda }$, λ is the laser wavelength used in the measurement of ΔΦ. Figure 8 shows the plot of the measured ΔΦ as a function of *I*_0_, which fits well to a second-order curve while also revealing a threshold behavior.

The properties of the polymerization process are largely determined by the parameters *η_p_*, σ_2_, and D_PI*_ in the coupled partial differential equation system. The polymerization coefficient *η_p_* is a parameter dependent on the specific combination of PI and monomer and determines how probable the polymerization is initiated, propagated, and terminated given a specific c_PI*_ and *c_DVB_*. The parameter σ_2_ reflects how efficient two-photon absorption happens to generate PI radicals. Finally, the diffusion constant D_PI*_ controls how far the PI radicals move around, which eventually determines the dimensions of the waveguides. The effect of D_PI*_ is clear when we compare poly-DVB waveguides with poly-phenylacetylene waveguides [24]. In the poly-phenylacetylene system, the small-molecule monomer (molecular weight 102 g/mol) also serves as a PI, which has a relatively larger D_PI*_ resulting in a waveguide width of 1.3 µm. In contrast, the OXE 02 used in this work as a PI is a much larger molecule (molecular weight 412 g/mol), so its D_PI*_ is much smaller yielding waveguides roughly 0.5 µm wide.

In a mixed PDMS/DVB system, the nonlinear refractive index is mainly of electronic origin, which usually falls within the order of 10^−15^ cm^2^/W. This would project to a refractive index change on the order of 0.001 at the writing focus. In contrast, the refractive index change after polymerization often saturates at the level of 0.1 (refractive index difference between the resulting polymer and the monomer) and increases over time during the laser irradiation. Thus, beam collapse and material damage can happen at any intensity above the polymerization threshold. This is well manifested in the fabrication of phenylacetylene waveguides. Indeed, the good quality of fabrication demonstrated in the present work benefits largely from the lower refractive index contrast. In theory, the self-focusing can be balanced with a well-controlled motion speed during fabrication, which is currently in practice. However, due to the nonlinearity the intensity can grow exponentially at small disturbances, and material homogeneity plays a key role in minimizing the fabrication defects.

Unlike poly-phenylacetylene, which features extensive conjugated carbon-carbon double bonds, poly-DVB is joined with mostly single bonds in the backbone. We thus expect that poly-DVB is mostly non-absorptive like polystyrene. In the waveguides constructed with poly-DVB, the transmission properties would be Rayleigh scattering-dominated in the visible band due to the molecular weight fluctuation in the resulting poly-DVB. This is precisely confirmed by the slope of −4 in the double-track waveguides in Figure 6b. The transmission loss of triple-track waveguides follows slope −4 in the shorter wavelength region but drops quicker than −4 in the longer wavelength region, which could indicate a constructive interference or resonance condition created by the more complex and larger structure. On the other hand, the high, achromatic transmission loss in the single-track waveguides suggests that their dimension may be below a threshold for low-loss transmission [35], with a significant mode area outside the waveguide core. It also further confirms the non-absorptive nature of poly-DVB in the relevant spectral band. Despite their lossy characteristics, we expect the single-track waveguides to be highly relevant and applicable in constructing photonic sensors.

In our experiments, the fabrication was performed in the open air with dissolved oxygen present in the system, which is a well-known polymerization retarder for styrene-type systems [36], although not an inhibitor. Oxygen is also a major quencher of many photoinitiators. This is manifested by the requirement of a relatively high concentration of PI during the fabrication and the much lower refractive index contrast compared with poly-phenylacetylene waveguides [24]. Thus, potentially significant improvements in the refractive index contrast may be attained through the use of nitrogen- or argon-purged DVB in an air-tight chamber during the fabrication, although the defects due to beam collapse will also be on the rise. This calls for a scheme of closed-loop laser intensity control based on the status of polymerization in the fabrication system.

## 5. Conclusions

We demonstrated the fabrication of submicron polymer waveguides in PDMS through two-photon direct laser writing. Our characterizations indicate a refractive index contrast of 0.005 and a relatively high transmission loss of 13 dB/cm on average, which is nearly achromatic within the spectral band of 535–679 nm. This confirms that poly-DVB is non-absorptive in the measured spectral band while also suggests that the dimensions of the waveguide are below a threshold for low-loss transmission. We further showed that the transmission properties can be significantly improved through the fabrication of compound waveguides, each of which consists of two- or three parallel laser-written tracks in close proximity (1.2 µm). Among the compound waveguides, the double-tracks show a Rayleigh scattering-limited transmission loss spectrum, ranging from 6.5 dB/cm at 535/43 nm to 2.2 dB/cm at 710/10 nm. The transmission loss spectrum of the triple-tracks deviates from a Rayleigh-scattering regime on the longer wavelength side, ranging from 12.4 dB/cm at 535/43 nm to 0.1 dB/cm at 710/10 nm. The deviation from the Rayleigh regime is possibly a result of constructive interference due to the larger and more complex refractive index structures in the triple-track waveguides.

## Figures and Tables

**Table 1:** Summary of transmission loss for the different waveguide types in different wavelength ranges in dB/cm.

Transmission Loss (dB/cm)			
Wavelength Range (nm)	Single-Track	Double-Track	Triple-Track
535/43	14.2 ± 2.9	6.5 ± 2.0	12.4 ± 0.3
561/14	13.5 ± 3.9	5.6 ± 1.7	10.8 ± 0.9
592/43	13.2 ± 3.4	4.5 ± 1.0	7.5 ± 0.7
609/54	12.6 ± 3.0	4.5 ± 0.9	6.6 ± 0.6
679/41	12.6 ± 2.3	2.6 ± 0.5	2.3 ± 0.4
710/10	-	2.2 ± 1.7	0.1 ± 0.4
